# A Retrospective Cohort Study on Predictors for Rehospitalizations With Recurrence of Atrial Fibrillation Post-Catheter Ablation for Atrial Fibrillation

**DOI:** 10.7759/cureus.16536

**Published:** 2021-07-21

**Authors:** Farhan A Shah, Nathan Mahler, Sean M Winkle, Priscilla Fujikawa, Brandon Nader, Juan Rodriguez

**Affiliations:** 1 Internal Medicine, LewisGale Medical Center, Salem, USA; 2 Internal Medicine, Edward Via College of Osteopathic Medicine, Blacksburg, USA; 3 Cardiology, LewisGale Medical Center, Salem, USA

**Keywords:** atrial fibrillation, catheter ablation, rehospitilzation, cardiac arrythmia, cardiac electrophysiology

## Abstract

Atrial fibrillation (AF) is the most common cardiac arrhythmia and is increasing in prevalence due to an aging population. Although medications for rhythm and rate control remain the first-line treatment options for many patients, difficulties can include arrhythmia relapse and drug side effects. Catheter ablation or radiofrequency is an alternative treatment modality that can isolate where ectopic arrhythmic sites originate. Several previous studies have examined post-ablation complications and hospitalization rates for arrhythmia recurrence. However, many of these studies used patient data from before 2015. We examined the following data using recent records: pre-procedural patient characteristics, rates of post-procedural hospitalizations with documented recurrence of AF, and patient risk factors associated with these recurrences.

## Introduction

Radiofrequency catheter ablation is a commonly used method for the treatment of tachyarrhythmias. Atrial fibrillation (AF) is the most common arrhythmia and has an increasing prevalence with our aging population [1]. While the use of medications for rhythm and rate control remains the first-line treatment for many patients, difficulties including rhythm relapse and drug side effects complicate their use. Radiofrequency is used to isolate the pulmonary vein, where most ectopic arrhythmic sites originate [2]. Post-procedural complications include hemorrhage, hematomas, pulmonary vein stenosis, recurrence of atrial fibrillation, cardiac tamponade, and hospitalization after ablation [3,4]. Various studies have looked at post-ablation complications and hospitalization rates for arrhythmia recurrence [5-7]. Many of these studies were conducted or used patient data from before 2015. Improvements in ablation techniques, and in our understanding of the mechanisms of AF, have led to improved patient outcomes. An important aspect of reducing the incidence of post-procedural complications is having current data regarding underlying patient factors that influence these complications [1]. In an effort to provide contemporary data regarding this patient population, we examined pre-procedural patient characteristics, rates of post-procedural hospitalizations with documented recurrence of AF, and patient risk factors associated with these recurrences.

## Materials and methods

The patient population studied ranged from age 40-85 years old, and was pooled from Hospital Corporation of America (HCA) facilities throughout the United States using HCA’s central database. Existing data from this source is de-identified and contains inpatient admission files including all diagnoses, procedures, and pharmacological data. We first identified all patients who underwent radioablation for atrial fibrillation in the year 2018 by using the Current Procedural Terminology (CPT) code of 93656. Data on age and gender were acquired. Patients were identified as specifically having a diagnosis of paroxysmal AF or persistent AF at the time of ablation based on having documented International Classification of Disease tenth edition (ICD-10) codes of I48.0 or I48.1, respectively. Pre-procedural patient medical histories, including cardiovascular medications, were obtained at the time of ablation using ICD-10 codes, family history queries, and home medication list queries. 

Medical histories were reviewed with particular emphasis on risk factors including cardiovascular risk factors (diabetes type 1 or type 2, hypertension, hyperlipidemia, and family history of heart disease), documented cardiovascular disease (heart failure with preserved or reduced ejection fraction, history of heart disease, and history of vascular disease), and other select pathologies (including chronic kidney disease, history of pulmonary embolism, or stroke). Prescribed cardiovascular medications that were being taken at the time of ablation were documented, as based on medication verifications performed by staff before the procedures. Medication groups included HMG CoA reductase inhibitors (HMGCRs), angiotensin-converting enzyme inhibitors (ACEI), angiotensin receptor blockers (ARB), vasodilators (including nitrates and hydralazine), beta-blockers, calcium channel blockers, thiazide diuretics, non-thiazide diuretics (including potassium-sparing and loop diuretics), platelet aggregation inhibitors, direct factor Xa inhibitors, and cyclooxygenase inhibitors (including non-steroidal inflammatory drugs and aspirin).

We then identified all patients who were hospitalized within 90 days and 365 days following their procedure who had documented recurrence of atrial fibrillation during that hospitalization based on the ICD-10 codes previously mentioned. These re-admitted patients were further analyzed based on their pre-procedural medical history, family history, and medications to determine possible associations between these characteristics and incidents of hospitalization with AF recurrence. Odds ratios were calculated with corresponding P values (significance cutoff of p < 0.05) and confidence intervals.

## Results

As seen in Table 1, we identified 9,189 patients who underwent radioablation for atrial fibrillation in 2018. The mean age was 66 years old and 63% of the patients were male. 50% of the patients had paroxysmal atrial fibrillation, 37% had persistent atrial fibrillation. Hypertension was a common co-morbidity, recorded in 47% of patients and 35% were being treated with an ACEI or an ARB, while 48% were on a beta-blocker. . Approximately 25% had a history of heart failure and diabetes. 73% of the patients were on anticoagulation and 24% were on a platelet aggregation inhibitor. 40% were on an HMG CoA reductase inhibitor.

**Table 1:** Baseline characteristics of the patients including patient demographics, comorbidities, and home medications shown as percentage. HMG: hydroxymethylglutaryl; ACEI: angiotensin-converting enzyme inhibitors; ARB: angiotensin receptor blockers; NSAIDs: nonsteroidal anti-inflammatory drugs.

Baseline characteristics of the patients (n = 9189)	
Age (years) – min-max (mean)	40-85 (66)
Male sex - no. (%)	5785 (63)
Female sex - no. (%)	3404 (37)
Paroxysmal atrial fibrillation - no. (%)	4571 (50)
Persistent atrial fibrillation - no. (%)	3366 (37)
Cardiovascular risk factors - no. (%)	
Hypertension	4343 (47)
Diabetes	2105 (23)
Hyperlipidemia	3398 (37)
Family history of heart disease	839 (9)
Chronic kidney disease	901 (9)
Cardiovascular disease - no. (%)	
Heart failure	2192 (24)
History of heart disease	330 (4)
History of vascular disease	135 (1)
History of pulmonary embolism and stroke	257 (3)
Home medications - no./total no. (%)	
HMG CoA reductase inhibitors	3653 (40)
ACEI	1760 (19)
ARBs	1496 (16)
Coronary vasodilators	318 (3)
Antihypertensives, vasodilators	532 (6)
Beta-blockers	4405 (48)
Calcium channel blockers	2228 (24)
Thiazide diuretics, ace inhibitor-thiazide or thiazide-like diuretic	757 (8)
Potassium sparing diuretics, combinations	57 (1)
Platelet aggregation inhibitors	2162 (24)
Direct factor Xa inhibitors	6713 (73)
NSAIDs, cyclooxygenase Inhibitors	1117 (12)

Table 2 illustrates the following:

90-day readmission for atrial fibrillation

In the 90 days following catheter ablation for atrial fibrillation, patients who were readmitted were more likely to be female (95% CI 1.037-1.376, p-value: 0.014), have heart failure (95% CI 1.25-1.789, p-value < 0.05), chronic kidney disease (95% CI 1.028-1.605, p-value: 0.027), be on HMG CoA reductase inhibitors (95% CI 1.069-1.499, p-value: 0.005), and on coronary vasodilators (95% CI 1.001-1.914, p-value: 0.049). On the other hand, those who were readmitted were less likely to be on an ARB (95% CI 0.659-0.986, p-value: 0.036).

365-day readmission for atrial fibrillation

Similarly to the 90-day readmission results, our 365-day analysis revealed that patients who were readmitted were more likely to have heart failure (95% CI 1.169-1.595, p-value < 0.05), HMG CoA reductase inhibitors (95% CI 1.049-1.364, p-value: 0.008), female gender (95% CI 1.088-1.388, p-value: 0.001), chronic kidney disease (95% CI 1.043-1.537, p-value: 0.017), ARBs (95% CI 0.699-0.986, p-value: 0.034). In addition, they were also more likely to be on platelet aggregation inhibitors (95% CI 1.067-1.407, p-value: 0.004) and have diabetes (95%CI 1.009-1.332, p-value: 0.037). At one year, patients readmitted had lower incidences of ARB treatment (95% CI 0.699-0.986, p-value: 0.034).

**Table 2:** 90- and 365-day readmission for atrial fibrillation according to comorbidities and home medications. HMG: hydroxymethylglutaryl; ACEI: angiotensin-converting enzyme inhibitors; ARB: angiotensin receptor blockers; NSAIDs: nonsteroidal anti-inflammatory drugs.

90- and 365-day readmission for atrial fibrillation						
	90 days			365 days		
	Odds ratio	p-value	95% CI	Odds ratio	p-value	95% CI
Heart failure	1.495	<0.05	1.25-1.789	1.365	<0.05	1.169-1.595
HMG CoA reductase inhibitors	1.244	0.005	1.069-1.449	1.196	0.008	1.049-1.364
Female	1.195	0.014	1.037-1.376	1.229	0.001	1.088-1.388
Chronic kidney disease	1.285	0.027	1.028-1.605	1.266	0.017	1.043-1.537
ARBs	0.806	0.036	0.659-0.986	0.83	0.034	0.699-0.986
Coronary vasodilators	1.384	0.049	1.001-1.914	1.32	0.054	0.995-1.752
Platelet aggregation inhibitors	1.005	0.948	0.853-1.185	1.226	0.004	1.067-1.407
Diabetes	1.127	0.146	0.959-1.325	1.159	0.037	1.009-1.332
History of pulmonary embolism, stroke	1.349	0.102	0.942-1.933	1.125	0.488	0.807-1.567
Hyperlipidemia	0.9	0.168	0.774-1.046	0.915	0.18	0.804-1.042
Family history of heart disease	1.167	0.173	0.934-1.458	1.085	0.418	0.891-1.321
History of heart disease	1.248	0.178	0.904-1.724	1.162	0.305	0.872-1.548
ACEI	0.899	0.252	0.748-1.079	0.907	0.226	0.775-1.062
Hypotensives, sympatholytic	0.693	0.262	0.364-1.316	0.851	0.527	0.516-1.404
NSAIDs	1.102	0.347	0.9-1.351	1.1	0.288	0.923-1.311
Vasodilators	0.869	0.364	0.643-1.176	1.016	0.901	0.793 - 1.301
Direct factor Xa inhibitors	1.068	0.414	0.912-1.249	1.048	0.494	0.916-1.2
Beta-blockers	0.943	0.424	0.817-1.089	0.977	0.715	0.863-1.106
Thiazide Diuretics	1.08	0.546	0.841 - 1.388	0.959	0.714	0.769 - 1.198
Hypertension	1.041	0.636	0.882 - 1.227	1.011	0.874	0.878 - 1.165
Calcium Channel Blockers	1.034	0.687	0.88 - 1.213	1.007	0.919	0.877 - 1.157
Adrenergic inhibitors	0.967	0.766	0.778 - 1.203	1.162	0.108	0.967 - 1.395
History of vascular disease	1.036	0.896	0.607 - 1.768	0.971	0.903	0.608 - 1.552

## Discussion

The use of catheter ablation for treatment of atrial fibrillation is a common and effective procedure that provides rhythm control where medication use has proved to be ineffective [8]. Medication failure has been defined as failure to adequately control the heart rate, or ongoing symptoms of palpitations, dizziness, and shortness of breath [9]. Ablation is most useful in patients with paroxysmal or persistent AF, but a growing body of evidence indicates that there may be mortality benefits to early use of rhythm control [10]. As a result, more consideration is being given to using ablation as first-line treatment in asymptomatic patients for rhythm control. Recurrence rates of AF after ablations have decreased dramatically with improved ablation technique, but remains a problem [11]. In this retrospective cohort study, we evaluated a total of 9189 patients who had a catheter ablation procedure for AF in 2018 at multiple institutions throughout the United States. We then identified patients who were re-hospitalized in the subsequent year with documented recurrence of atrial fibrillation. Overall, 1,533 patients (16.68%) were readmitted within the first 90 days, of whom 959 (10.4%) were found to have recurrent AF. 4306 (46.86%) patients were readmitted within 365 days, of whom 1,363 (14.83%) were found to have recurrent AF. Recurrence of AF in the first 90 days is common and often not defined as true ablation failure, referred to as a “blanking period” [12]. Not all patients with an early event go on to develop recurrence, however the two are associated to some degree [13]. More recent studies have shown 90 day recurrence rates of 28% [14] and one year recurrence rates of 27.6% [1]. Our data does not reflect outpatient, emergency department, or heart monitor detection of recurrence. However, comparison of similar older studies documenting hospitalization for AF recurrence in one year suggest that our 14.83% recurrence rate is an improvement from previously recorded and reported rates. [15]. Advancement in catheter design and accuracy of target mapping in recent years has led to improved outcomes as reflected by our data [1]. Of note, recurrence rates in persistent AF are higher than paroxysmal, and half of the patients in our study had paroxysmal AF, which may have skewed our data by an unknown amount [16]. Of our reviewed population, 13% were not specifically indicated paroxysmal nor persistent AF, which is likely due to documentation error or patients with long-standing persistent AF in whom ablation is sometimes attempted.

 Various factors have been associated with higher post-procedural AF recurrence rates. We evaluated patients based on history of cardiovascular diseases, cardiovascular risk factors, and medications being taken at the time of ablation. In congruence with prior studies [17], we found an increased risk of AF recurrence associated with female gender, chronic kidney disease, and heart failure in both the 90- and 365-day period. Despite increased prevalence of AF in women, it has been observed that fewer women undergo catheter ablation and timing of ablation after onset may be delayed. It is likely that both social and gender-specific attributes play a role in these trends [18]. It has been reported that female gender is an independent risk factor for AF recurrence, but other studies show that female gender is protective against recurring AF [11]. More research is needed to both understand and improve outcomes in this area. Heart failure is also an important area of focus regarding ablation. AF is a common comorbidity in patients with heart failure. Recurrence rates after single ablation are known to be higher than in patients without heart failure [19]. We were unable to characterize heart failure by ejection fraction or type (diastolic vs. systolic), but our findings reaffirm that heart failure is a risk factor for AF recurrence after first-time ablation. Chronic renal impairment was also a significant risk factor in our study, which has also been noted in previous studies [20]. 

 Some variables that were statistically significant risk factors for AF recurrence in previous studies did not show significance in our study, including hypertension. This may be due to the lack of an outpatient data contribution or the duration of our study. Notably, other studies have only found hypertension to be significant in a two-year follow-up period [11]. Diabetes was a significant factor in the 365-day recurrence group but not in the 90-day readmission group. Metabolic syndrome has been found to have a similar pattern in other studies [12]. Hyperlipidemia was not a significant risk factor for recurrence in our study. Interestingly, the use of HMGCRs was significantly associated with increased recurrence rates, in our study. Hyperlipidemia was possibly underreported in our patient group, as it is noted that 45.25% of patients in the 90-day readmission group were taking HMGCRs while only 37.74% had documented hyperlipidemia. A similar discrepancy was seen in the 365-day readmission group. We conclude that hyperlipidemia is likely a significant risk factor for recurrence in this study, as the accurate number of patients with hyperlipidemia is likely reflected by the number of patients who were on statin therapy. Statin therapy may not reduce the impact of hyperlipidemia. Prior studies have found elevated low‐density lipoprotein cholesterol to be a significant risk for AF recurrence, but saw no association of statin therapy and improved recurrence rates [21]. Further data is needed, including trended cholesterol levels, length of statin use, and potency of the statin used. Other medications of significance included coronary vasodilators and ARBs. Vasodilators (including nitroglycerine) were associated with increased AF recurrence risk, which may reflect that this population was more likely to have coronary artery disease. Lastly, we note that ARBs were associated with a decreased risk of AF, but ACEIs were not. More data is needed regarding dosing and compliance of these medications before a substantial conclusion could be drawn regarding a protective role of ARBs in the prevention of recurrence.

Some limitations to the study centered around difficulty with defining specific parameters for risk factors when using a large patient database. Specific data reflecting heart failure type may assist with defining which heart failure populations that are at greatest risk for recurrence. Specific data regarding diabetes, including A1cs and diabetic type may show impact of treatment on that patient group.

## Conclusions

Other studies have shown an important relationship between duration of AF prior to ablation and recurrence rates. Confidently attributing recurrence to AF duration is difficult given high variation in reporting of duration prior to procedures. There may be some risk factors that are only of significance in patients with persistent AF, which would require a further sub-analysis of patients with paroxysmal versus persistent AF. Studies of post-ablation outcomes are extremely varied in their selection of patient age, comorbidities, procedure type, which is an added challenge when comparing findings of other institutions. This cohort study should prompt further examination of post-catheter ablation complications and rehospitalizations in other populations and healthcare systems. 
